# To Junior Dentists

**Published:** 1886-07

**Authors:** 


### TO JUNIOR DENTISTS. No. VI.

#### THE PREPARATION OF CAVITIES FOR FILLING.

*Dear Doctor :*

Since my return from the excursion on which I made the visit to your office and witnessed the operation which you requested me to criticise freely and fully, I have been considering the propriety of doing so, and have finally concluded to take the risk. It is usually a very hazardous thing to undertake to point out the faults of a friend. Do you remember the story of Gil Blas and the Archbishop? You will, perhaps, remember that Gil Blas was engaged as secretary by the latter, under the strictest injunction to inform his master if, in the progress of age, he began to detect any gradual weakening of the intellectual powers, "for," said the Archbishop, "I do not wish to detract from my fame by lingering in the pulpit when my powers of reasoning shall have failed." In the course of time, when the ecclesiastic had for a long period exhibited his senility to every one, Gil Blas ventured on hinting to him that it was time that he retired, and was instantly dismissed, with the intimation that it was his own judgment that was lacking, and that the Archbishop was fully satisfied that his mental powers were never so vigorous. Yet despite such warnings, I now propose to point out as plainly as I can the defects that I saw in your operation. I could not do this at the time, because I would not let your patient know that the fillings were imperfect.

As I look back down the vista of the years that are gone, and call to mind the time when I too was a young dentist, I remember with what complacency I contemplated my early operations. I had not sufficient experience at that time, and was too little of an operator to be able to see the faults that existed. Like most young graduates, I was entirely convinced of my own great abilities, and felt that it was my mission to lift operative dentistry to a higher plane. I affected to despise the mechanical branch of my business, though I worked diligently enough at it when I had any of it to do, for it was upon that I was chiefly dependent for bread and butter. I inserted fillings and spent hours in polishing them up, in burnishing the surface and admiring the shining gold. You can imagine my consternation and horror when some of those very fillings came to me shortly after, brought in the hand of the patient, and when about others I saw the fatal blue line that indicated failure. Well, I have spent many a sleepless hour, fairly blushing in the solitude of my chamber, in mortification and humbled pride at the miserable defects of that which I had regarded with so much complacency. It was a bitter thing to be convinced that I was not half so fine an operator as I had imagined myself, and instead of pursuing my triumphant course to be obliged to painfully retrace my steps and laboriously to search for the causes of my failures. I found some of them, and having rectified these, again began to plume myself upon my attainments, only again to be humbled by new failures, and once more to creep painfully back to the beginning in search of the source of my humiliation. And this has been about the history of my professional life, and there is little doubt that it will be yours. You may make less mistakes than I have made, but when you arrive at middle life there will be, if you are the honest man that I think you are, little comfort in looking back over much of your professional life. Your chief satisfaction will be that, despite your stumblings, you have yet made steady progress.

When I intimate that the fillings that you inserted with so much pride, while I was visiting you, will prove failures within a comparatively brief time, you will be shocked, and perhaps indignant. Wait for ten or fifteen years, and it will not then seem to you such a matter of surprise that you should be thought capable of errors. You will have lost one-half of your conceit, but you will have gained in solid judgment.

You remember that the operation was the filling of cavities in the distal approximal surface of a central, and the adjoining surface of the lateral incisor. I doubt very much whether, in the preparation of the cavities, you paid the remotest attention to the structure, character and contour of the teeth themselves, and yet not unfrequently this should have much to do in guiding your course. The patient was a young lady, with teeth of a bluish-white color, the enamel very thin and as fragile as glass. Now that meant that the edges should be most carefully protected. A filling, simply flush with the margins, is not enough in such cases. More is required. Such an operation demands plenty of space. Highly polished gold, very conspicuously displayed in such teeth, is extremely offensive to every person of taste. What was your method of procedure?

In the first place, you had gained no space in advance of the operation. You depended upon rapid wedging, and when you came to try this you found, as you should have anticipated, that in such a mouth, with such a patient, one who had fully reached maturity and whose osseous tissues gave every evidence of firmness and fineness and closeness of texture, it was impossible to gain sufficient room for the kind of operation demanded. Then, instead of dismissing her with a wedge between the teeth until you could correct the first blunder, you proceeded to operate with the odds against you. That, my boy, was your second error. I fear that you will make many failures before you learn this important lesson:

*No man who does first-class work will commence until he is master of the field and can control all the environments.*

You made another mistake at the outset. You commenced your operation without first putting on the rubber dam. As a consequence, your wedges had mostly to be withdrawn before you could begin the filling. You did most of the excavating when the cavity was flooded with moisture, and when it was impossible to see precisely what you were doing. Besides, you subjected your patient to unnecessary pain.

*No good dentist is forgetful of the comparative comfort of his patient.*

Before you had finished the excavation of the central you found that you had not entire command of the case, for you had undermined the anterior wall of the cavity to such an extent that it would have been impossible to perfectly fill it had it not crumbled away. You had previously chiseled it out freely, to get the room that should have been gained by preliminary wedging, and you now had a yawning chasm, with no front wall. You then inserted a bur in the engine, and proceeded to gain anchorage beneath the enamel above and below the cavity. I knew what would be the consequence when I saw you put in that large and sharp bur. You undermined more of the glassy enamel, and fractured it, thus increasing the size of the already too large cavity. There was no decay to be removed at that point, and you needed but a minim bur, that your anchorage might be in the dentine. Better yet would it have been, had you employed only an excavator. You did not consider the character of the tooth that you were at work upon. Then, too, your attention was distracted by your attempts to hold conversation with me, and by constant directions to your office boy concerning something that was going on in the laboratory.

*No man can do two things at once. To perfectly fill a bad cavity demands all the resources of both mind and body.*

Finally, when you had finished the excavation of the central, you had a cavity that was fully twice as large as it should have been, for much of sound tooth structure had been unnecessarily sacrificed. Had you been at work on a tooth of a different character, there might have been danger of insufficient excavation, but with such a tooth as the one that you were then to operate upon, the danger was from too much cutting away.

I watched you keenly, to see if you had learned anything in the excavation of the first tooth. I will do you the justice to say that you had one thing impressed upon your mind, and that was to be careful in undermining those fragile walls. But it led you to a fault in the other direction. You were now at work upon a smaller tooth than the central, yet the cavity was quite as large. What should have been the inference ? That greater care was required in the excavation, because the distance to the pulp was considerably less. Yet you repeated the error of the previous cavity by inserting the same engine bur, which was too large for even the central. To avoid the crumbling which succeeded a too near approach to the thin enamel, you buried the bur in the dentine, and I was momentarily expecting to see you cut some filament of the pulp, and thus have another difficulty to contend with. You may thank your lucky stars that the patient was not a few years younger, or that the pulps lay unusually deep in her teeth.

*The judicious operator, while avoiding one extreme, is careful not to go to the other.*

You succeeded in finishing the excavation of this cavity without wounding the pulp, and without inducing such extensive crumbling of the enamel as in the first tooth. But your anchorage was too deep. Indeed, you burred so near the cutting edge of the tooth in your anxiety to make a sure thing of it, that the breaking off of the point thus weakened is only a question of time. Then what will you do with such a fragile tooth as is that one? When you finally placed the rubber dam in position, I was in a maze of wonderment to know if you called the excavating and preparation of the cavity finished. You certainly did, for immediately upon drying the cavity you began to insert the gold. And now let me point out to you what the condition was. I have already called your attention to the thin, glassy, fragile character of the enamel. If you had examined it with a good magnifying glass you would have seen how rough and serrated were the edges. It was absolutely essential that these be made smooth, if a perfect filling was to be inserted. A fine stone should have been used to dress down these ragged margins, and fine emery strips should have polished them to a glossy smoothness, leaving the margin slightly beveled toward the exterior.

*It is impossible to obtain perfect contact with a rough and craggy surface.*

As it was, you proceeded to introduce your filling upon a series of sharp and easily fractured points. Under the blows of the mallet these must have crumbled, leaving a yet rougher and more imperfect edge, with the margin of the gold all exposed at innumerable minute points. The glassy edges, instead of being protected with an overlapping layer of gold carefully impacted, were left in that imperfect condition, to continue the process of disintegration. What is to be expected from such an operation but early failure, with consequent mortification to you and loss to the patient?

I was curious to see if you would take any measures to protect the pulp of the lateral from thermal changes, but you did not. I think that a temporary filling of gutta-percha or oxy-phosphate of zinc should have been inserted, until the immediate danger of pulpitis should have passed away. I noticed that when at the completion of the operation you gave the young lady some water with which to rinse her mouth, she flinched very sensibly. I predict trouble there, if it has not already occurred.

*A sensitive tissue, like the pulp of a tooth, may not be subjected to rude shocks, long continued, with impunity.*

Then, too, you had not been sufficiently careful in leaving proper support for the anterior wall of enamel in the lateral. It was impossible to perfectly fill the deep undercut which you had made, and the enamel was unnecessarily weakened. Both of the fillings must prove failures, within a short time. There were faults in the insertion of the gold, but I do not propose now to advert to them. Indeed, I think I have said all that your friendship for me will bear. It is quite likely that you will feel indignant, and entertain the idea that I have purposely exaggerated the defects. If you will think it over candidly, however, you cannot but admit the probable justice of my strictures. Let it be a lesson to you to avoid these mistakes in the future.

*No error is to be wholly deplored if it teaches us to avoid such faults in the future.*

